# Generation of human induced pluripotent stem cell lines derived from patients of cystic biliary atresia

**DOI:** 10.1007/s13577-024-01147-x

**Published:** 2024-11-13

**Authors:** Ningxin Ge, Kan Suzuki, Iori Sato, Michiya Noguchi, Yukio Nakamura, Mami Matsuo-Takasaki, Jun Fujishiro, Yohei Hayashi

**Affiliations:** 1 iPS Cell Advanced Characterization and Development Team, BioResource Research Center, RIKEN, 3-1-1 Koyadai, Tsukuba, Ibaraki 305-0074 Japan; 2 School of Integrative and Global Majors, University of Tsukuba, 1-1-1 Tennodai, Tsukuba, Ibaraki 305-8577 Japan; 3 Division of Pediatric Surgery, Surgical Oncology Graduate School of Medicine, Dokkyo Medical University, Tochigi, Japan; 4 Cell Engineering Division, BioResource Research Center, RIKEN, 3-1-1 Koyadai, Tsukuba, Ibaraki 305-0074 Japan; 5 Department of Pediatric Surgery, The University of Tokyo Hospital, 7-3-1 Hongo, Bunkyo-ku, Tokyo, 113-8655 Japan

**Keywords:** Human induced pluripotent stem cells, Biliary atresia (BA), Self-renewal, Pluripotency, Whole genome sequencing

## Abstract

Biliary atresia (BA), resulting from abnormal development of the liver’s internal or external bile ducts, can lead to liver damage and potentially fatal cirrhosis. Type I cystic biliary atresia is a relatively uncommon, but clinically significant variant of BA. It is critical to develop experimental models of BA to examine the etiology and pathogenesis, which remain elusive, and to develop future therapeutics. Here, we have successfully generated a panel of human induced pluripotent stem cells (hiPSCs) from five Japanese patients carrying type I cystic BA. These hiPSC lines exhibited characteristics of self-renewal and pluripotency. These cells held normal karyotypes mostly, but one of them carried hemizygous deletions, the clinical significance of which is unknown yet. Whole genome sequence analysis indicated that some of the mutations or single nucleotide polymorphisms (SNPs) commonly found in these patients are related to hepatobiliary abnormality. Given the limited understanding of the molecular pathogenesis of cystic BA, attributed to unknown factors of genetic and environmental causes, these cellular resources will be instrumental in replicating disease phenotypes and in advancing novel therapies for this disease.

## Introduction

Biliary atresia (BA) is an obstructive fibrotic cholangiopathy of the intrahepatic and extrahepatic bile ducts that causes pathological jaundice and liver failure in early infancy. Even if surgical removal of extrahepatic bile duct remnants and subsequent hepatoenterostomy are successful, most patients are likely to present with progressive liver dysfunction. It is the most common indication for pediatric liver transplantation [1]. Further improvement in outcome will require a greater understanding of the mechanisms of biliary injury and liver fibrosis. There are currently no convincing theories regarding the cause of BA, including abnormal organ development, viral infection, genetic predisposition, and maternal immunity. Thus, to elucidate the pathogenesis of BA and to develop alternative therapeutics, sophisticated disease models are required.

Animal models of BA have been generated with several methods. Heterozygous deletion of the *SRY- related HMG-box 17 (Sox17)* gene in mice exhibits a condition like BA [2–4]. *Pkd1l1*-deficient mice and zebrafish exhibited bile duct hypertrophy, reduced biliary drainage, and liver fibrosis [5–7]. Mice infected with rotavirus displayed symptoms similar to human biliary atresia, with bile duct obstruction, bile duct proliferation, and liver inflammation with fibrosis [8]. Also, a plant toxin, biliatresone, has been shown to induce BA-like symptoms in several animal models [9–11]. Neonatal BALB/C mice injected with biliatresone developed clinical signs of biliary obstruction, and dysplasia or the absence of extrahepatic biliary tract lumen, which confirmed the occurrence of BA [12]. In another study, neonatal C57BL/6 J mice exposed to biliatresone exhibited clinical symptoms of BA, such as jaundice, twisted and enlarged extraphepatic bile ducts, and cholestasis [13]. Also, the pups of mice treated with low-dose biliatresone during pregnancy have altered bile acid and immune profiles similar to those observed in BA patients, even in the absence of significant histological changes [14]. These studies demonstrate that biliatresone-treated mice are useful for modeling BA and may serve as a tool for exploring therapeutic interventions. However, it is unclear whether these models reflect the natural pathogenesis of human BA or not [15, 16].

Research on samples and data from BA patients is only possible after the onset of the disease [17, 18]. Recent studies have focused on the potential of BA-specific human liver organoids as research models. Biliary organoids derived from liver biopsies of BA patients revealed molecular and functional evidences of delayed epithelial development in BA patients [19]. Human liver organoids treated with a synthetic immunostimulant that is structurally similar to a double-stranded RNA (dsRNA) found in the reovirus and rotavirus, polyinosinic:polycytidylic acid (poly (I:C)), exhibited morphology and genetic signature highly compatible to organoids developed from BA liver samples [20]. Also, human liver organoids treated with biliatresone exhibited a decrease in cell–cell tight junctions, polarity changes, increased epithelial permeability, and loss of cilia and cilia function in cholangiocytes [21]. These studies demonstrate that human liver organoids from BA patients or chemical treatments are excellent disease models to recapitulate the symptoms of BA. In contrast, building an experimental model that can monitor the bile duct damage process during development will lead to further elucidation of the pathogenesis of BA. Patient-derived human induced pluripotent stem cells (hiPSCs) can be promising tools to recapitulate the pathogenesis during development. Indeed, only one pioneering study reported that BA-specific hiPSCs showed deficiency in biliary differentiation along with increased fibrosis, the two key disease features of BA to date [22]. To expand this report to the general conclusions of the modeling of BA, more hiPSC samples are required since the genetic cause of BA is unknown, and the ethnicity and geographical distribution of the epidemiology of BA may be variable [23, 24].

Here, we have established type I cystic BA-specific hiPSC lines from five Japanese female patients and performed whole genome sequencing (WGS) on these samples. Type I cystic BA is a relatively uncommon, but clinically significant variant of BA. These hiPSC lines derived from type I cystic BA patients should contribute to the development of pathological models and the elucidation of the molecular pathogenesis of cystic BA.

## Results and discussion

We collected PBMCs (peripheral blood mononuclear cells) from five patients of type I cystic BA patients. The general clinical information of these patients is shown in Table 1. All the patients were diagnosed soon after birth and performed Kasai surgery operation. These patients were not associated with Alagille syndrome or Hajdu–Cheney syndrome because they did not develop any symptoms other than hepatobiliary systems. Also, morphologies of the common bile duct in these patients show cystic dilatation, which differs from hypoplasia observed in Alagille syndrome patients. No liver transplantation was performed on these patients until the collection of PBMCs. The hiPSC lines derived from these cystic BA patients (cBA-hiPSCs) were generated using episomal plasmid vectors from these PBMCs [25, 26].

**Table 1:** Summary of general clinical information of cystic type I BA patients recruited for this study

Patient number	HiPSC line name	Age at blood collection (years)	Sex	Ethnicity	Age at Kasai surgery operation (days)
1	HiPS-cBA1	3	Female	Asia	59
2	HiPS-cBA2	6	Female	Asia	64
3	HiPS-cBA3	25	Female	Asia	62
4	HiPS-cBA4	28	Female	Asia	37
5	HiPS-cBA5	5	Female	Asia	28

**Fig. 1:**
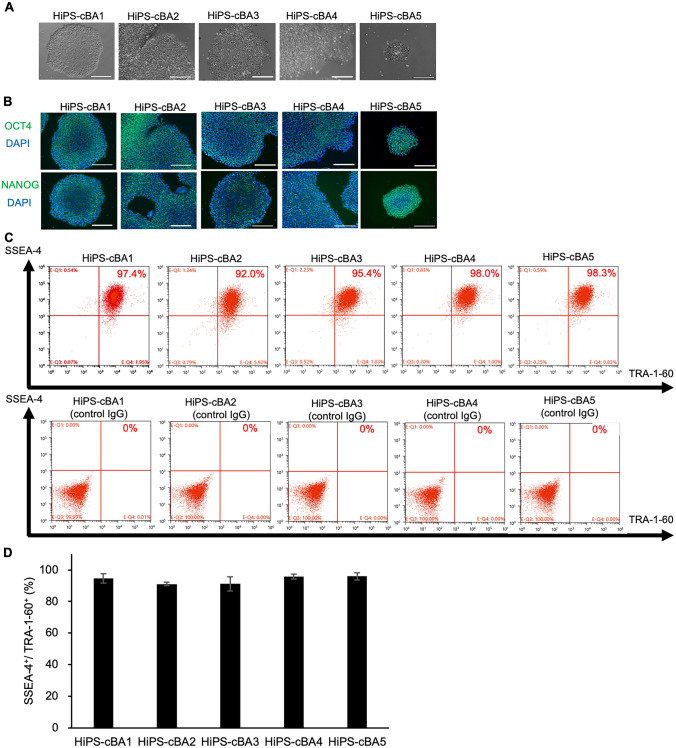
Self-renewal marker expression of hiPSC lines derived from cystic BA patients. **A** Morphologies of BA-specific hiPSCs taken with phase contrast microscopy. Scale bars, 200 µm. **B** Immunocytochemistry of OCT3/4 and NANOG on cBA-hiPSCs. Scale bars, 200 µm. **C** Flow cytometry analysis of SSEA-4 and TRA-1-60 on cBA-hiPSCs. Top panels: samples with anti-SSEA1 and anti-TRA-1-60 antibodies. Bottom panels: samples with control IgG. **D** The ratio of positive cells for SSEA-4 and TRA-1-60. The bar graph shows mean + standard errors (SE), *n* = 3 (biological samples)

The generated cBA-hiPSC lines were cultured in an undifferentiated state and characterized to confirm self-renewal capacity and pluripotency. All the cBA-hiPSC lines formed typical human embryonic stem cell (hESC)-like colonies under feeder-free culture conditions (Fig. 1A). The expression of the self-renewal markers, OCT3/4 and NANOG, was detected in these cBA-hiPSC lines with immunocytochemistry (Fig. 1B). The expression of cell surface markers, SSEA-4 and TRA-1-60, was also detected with flow cytometry (Fig. 1C). All the cBA-hiPSC lines contained cells more than 90% positive for SSEA-4 and TRA-1-60 (Fig. 1D).

**Fig. 2:**
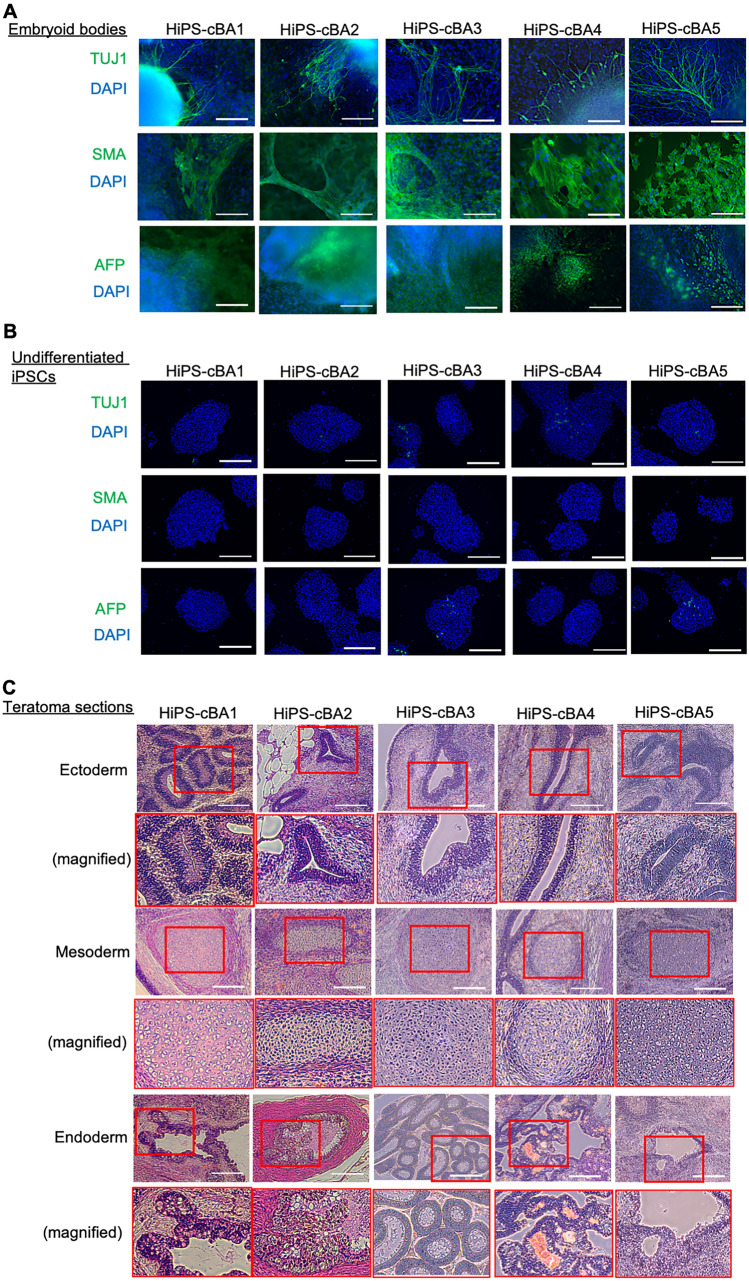
Pluripotency of hiPSC lines derived from cystic BA patients. **A** Immunocytochemistry of TUJ1, SMA, and AFP on embryoid bodies (EBs) differentiated from BA-specific hiPSCs. Scale bars, 200 µm. **B** Immunocytochemistry of TUJ1, SMA, and AFP on undifferentiated cBA-hiPSCs. Scale bars, 200 µm. **C** Sections with hematoxylin and eosin (HE) staining on teratomas derived from cBA-hiPSCs. Neural rosettes, cartilages, or intestine-like secretion tissues are indicated as derivatives of ectoderm, mesoderm, or endoderm, respectively. Red rectangles are magnified regions. Scale bars, 200 µm

**Fig. 3:**
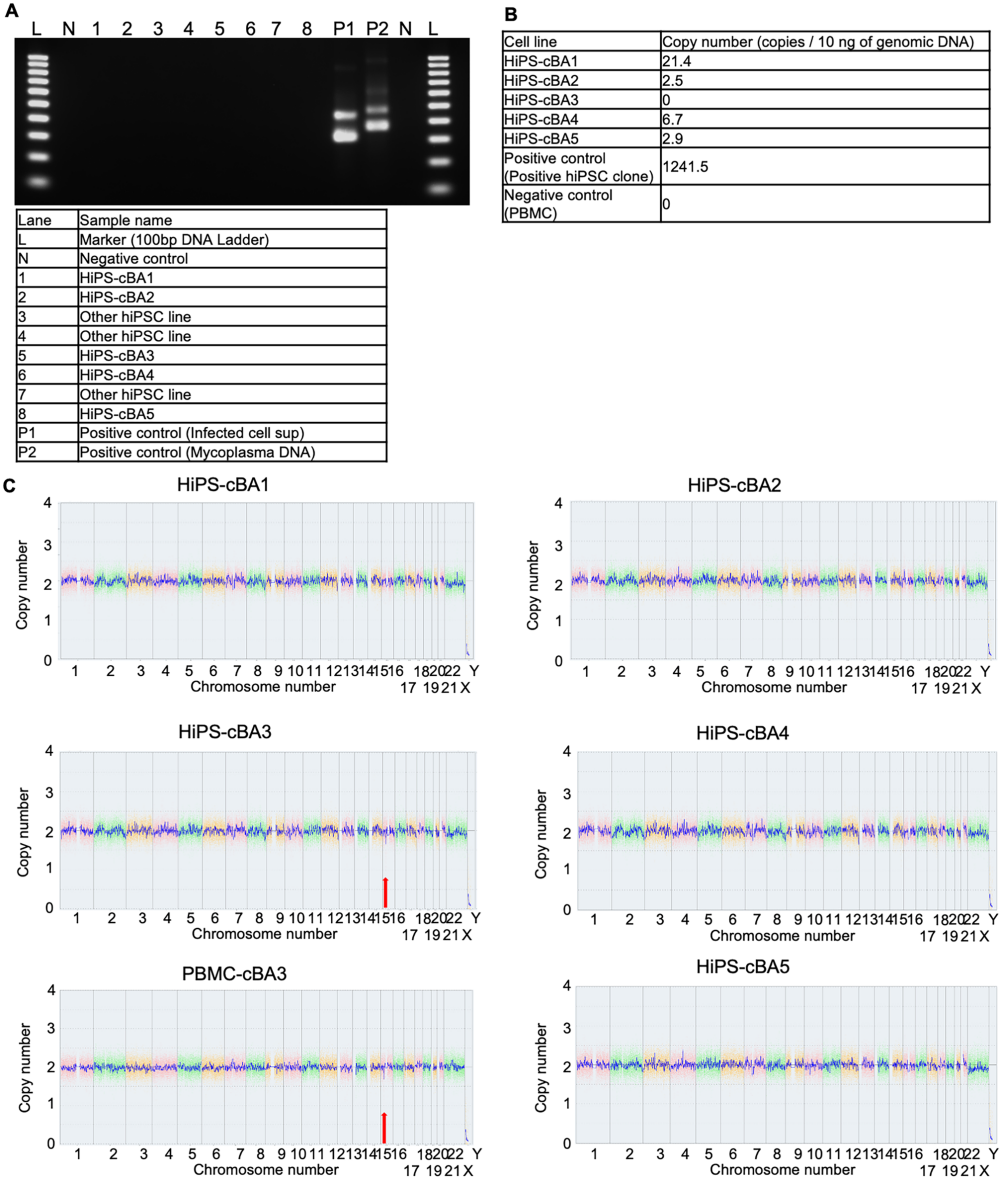
Characterization of hiPSC lines derived from cystic BA patients. **A** Mycoplasma test using PCR on conditioned medium samples. **B** Episomal vector test using PCR on genomic DNA samples. **C** Copy number analysis from CGH array on genomic DNA samples. Red arrows indicate heterozygous deletion of the 16p12.2 region

The pluripotency of these cBA-hiPSC lines was assessed using embryoid body (EB) formation and teratoma formation assays. Immunocytochemistry for an ectodermal marker, TUJ1 (TUBB3; tubulin, beta 3 class III), a mesodermal marker, SMA (smooth muscle actin), and an endodermal marker, AFP (α-fetoprotein), demonstrated that EBs generated from these cBA-hiPSC lines contained differentiated cells from all three germ layers (Fig. 2A and B). Teratomas derived from these lines contained tissues from all three germ layers, such as neuroepithelium or melanocyte (ectoderm), cartilage (mesoderm), and gastrointestinal-like structures (endoderm) (Fig. 2C). All cBA-hiPSC lines tested negative for mycoplasma contamination (Fig. 3A). The episomal plasmid vectors used for generating hiPSCs were confirmed to be absent or little in the genomic DNA of all cBA-hiPSC lines by qPCR targeting of the EBNA1 sequence (Fig. 3B). CNV (copy number variation) microarray analysis revealed that these lines had normal karyotypes, except for a heterozygous deletion of 16p12.2, which includes the METTL9, IGSF6, and OTOA genes in HIPS-cBA3 (Fig. 3C). This deletion was also found in the original PBMCs from the same patient. This large deletion is considered potentially pathogenic, especially for causing hearing loss when both OTOA alleles are disrupted [27, 28]. However, whether this deletion is linked to biliary atresia remains uncertain.

**Fig. 4:**
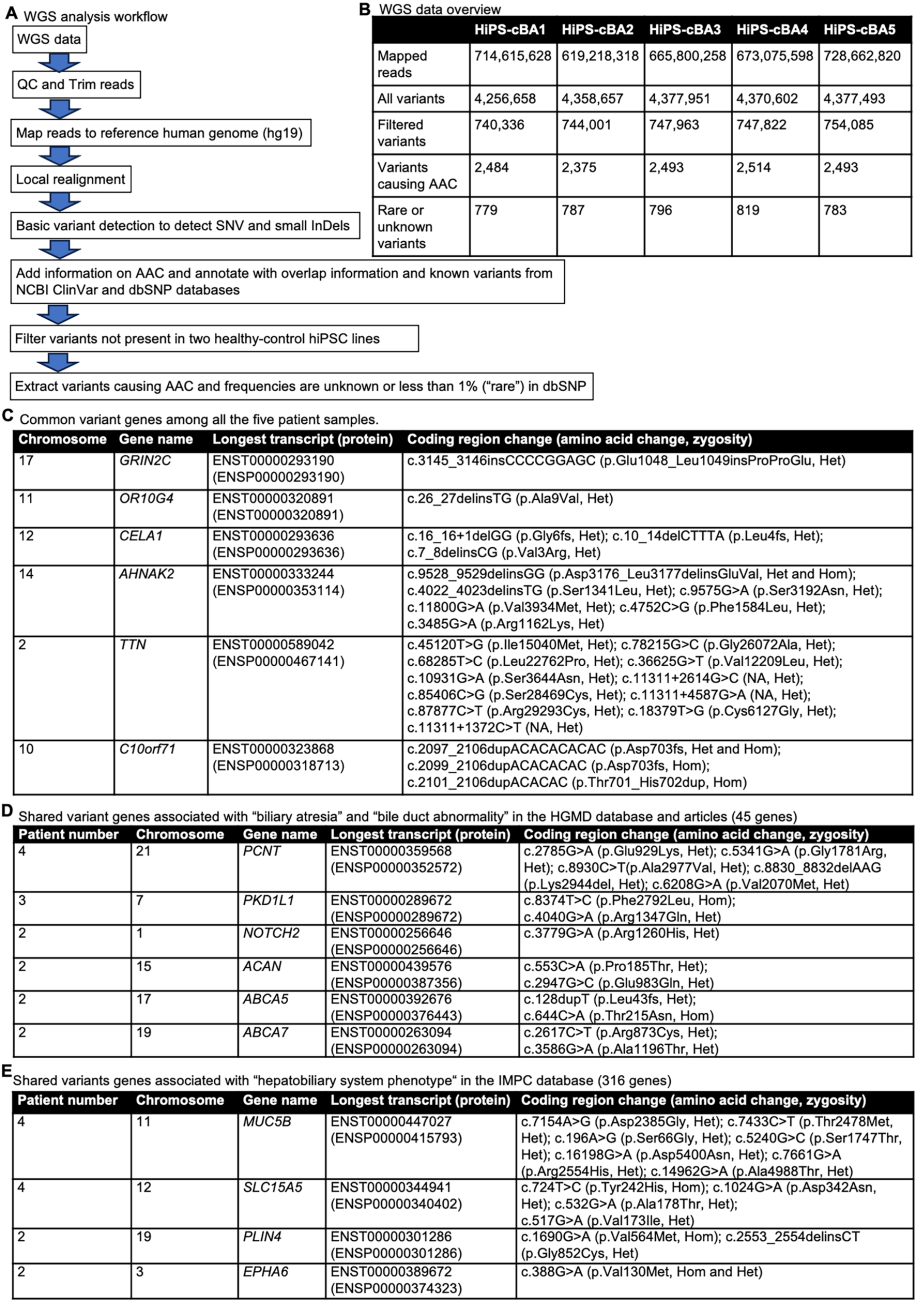
Whole genome sequencing (WGS) analysis of cystic BA patients. **A** Workflow of WGS analysis. QC: quality check, hg19: human genome assembly, Genome Reference Consortium (GRC) h37, SNV: single nucleotide variant, InDels: insertions or deletions, AAC: amino acid changes, and SNP: single nucleotide polymorphism. **B** Summary of WGS and variant numbers in the analysis of each sample. **C** Common variant genes among all the five patient samples. Chromosome numbers, gene names, longest transcript and its proteins in Ensembl format, coding region change with amino acid change, and zygosity are shown. **D** Shared variant genes associated with “biliary atresia” and “bile duct abnormality” in the HGMD database and articles (45 genes). Patient numbers, chromosome numbers, gene names, longest transcript and its proteins in Ensembl format, coding region change with amino acid change, and zygosity are shown. **E** Shared variant genes associated with “hepatobiliary system phenotype" in the HGMD database and articles (316 genes). Patient numbers, chromosome numbers, gene names, longest transcript and its proteins in Ensembl format, coding region change with amino acid change, and zygosity are shown

WGS was performed on these cBA-hiPSC lines. The workflow of the analysis is shown in Fig. 4A. From each sample, 740,000–750,000 variants were obtained from 610 to 730 million mapped reads (Fig. 4B). After filtering variants with two healthy control hiPSC samples and selecting those causing amino acid changes (AAC) with a frequency of less than 1% (considered "rare") or unknown, approximately 800 variants were identified in each sample. Common variant genes found in all patient samples included *GRIN2C*, *OR10G4*, *CELA1*, *AHNAK2*, *TTN*, and *C10orf71*; however, no published studies have linked these genes to hepatobiliary abnormality (Fig. 4C). Next, we extracted variant genes shared by two or more patients and cross-referenced them with previous studies and the Human Gene Mutation Database (HGMD) [29] to identify those potentially associated with bile duct abnormalities. In this category, *PCNT (pericentin)*, *PKD1L1 (polycystic kidney disease 1-like 1)*, *ACAN (aggrecan)*, *ABCA5 (ATP-binding cassette subfamily A member 5****)***, *ABCA7*, and *NOTCH2* were identified (Fig. 4D). *PCNT* was identified as a de novo mutation in BA patients, and PCNT knockout zebrafish exhibited reduced biliary flow [30]. Variants in *PKD1L1* genes were also identified to be associated with BA in several studies [31, 32]. *Pkd1l1*-deficient mice and zebrafish showed abnormal phenotypes related to BA [5–7]. NOTCH2 is primarily a gene responsible for Alagille or Hajdu–Cheney syndrome, but may also be implicated in BA etiology [32]. ACAN, ABCA5, and ABCA7 have not been specifically linked to BA, but further research would be needed to clarify their roles. We also extracted variant genes shared by two or more patients and cross-referenced them with genes associated with "hepatobiliary system phenotypes" from the International Mouse Phenotyping Consortium (IMPC) [33]. In this category, *MUC5B (mucin 5B)*, *SLC15A5 (solute carrier family 15 member 5)*, *PLIN4 (perilipin 4)*, and *EPHA6* were identified (Fig. 4E). MUC5B is specifically expressed in gallbladder [34, 35], and homozygous Muc5b-deficient mice have smaller livers (https://www.mousephenotype.org/data/genes/MGI:1921430). SLC15A5 is a relatively uncharacterized gene, but homozygous knockout mice exhibit enlarged gallbladders, a sign of cholecystitis, according to the IMPC database (https://www.mousephenotype.org/data/genes/MGI:3607714). Knockout mice for *PLIN4* or *EPHA6* show abnormal liver morphology (https://www.mousephenotype.org/data/genes/MGI:1929709, https://www.mousephenotype.org/data/genes/MGI:108034). In summary, we identified interesting variants in genes potentially related to BA or hepatobiliary abnormality in these cBA-hiPSCs, although the impact of these variants on protein functionality remains unclear. These findings suggest that BA is not a monogenic disease but rather polygenic, or it could be associated with viral perinatal infections, toxins, and immune dysregulation. Notably, the *PCNT* and *PKD1L1* genes are involved in cilia formation, and cilia dysfunction may be a key molecular mechanism underlying BA pathogenesis [5, 21].

In this study, we successfully generated five cBA-hiPSC lines with WGS information. These hiPSC lines derived from type I cystic biliary atresia patients should contribute to the development of pathological models and the elucidation of the molecular pathogenesis, since the genetic and/or environmental factors to cause BA have not been fully elucidated.

## Materials and methods

### Establishment and culture of hiPSCs

The hiPSCs were generated from PBMCs obtained from BA patients using episomal plasmid vectors to express *OCT4, SOX2, KLF4, L-MYC, LIN28, mp53DD, and EBNA1* (Epi5 episomal iPSC reprogramming kit; A15960, Thermo Fisher Scientific) [25, 26]. The resulting cBA-hiPSCs were designated as HiPS-cBA1 (BRCi028-A in hPSCreg), HiPS-cBA2 (BRCi029-A in hPSCreg), HiPS-cBA3 (BRCi032-A in hPSCreg), HiPS-cBA4 (BRCi033-A in hPSCreg), and HiPS-cBA5 (BRCi035-A in hPSCreg). For seeding cBA-hiPSCs at every passage, StemFit AK02N medium (Ajinomoto, Tokyo, Japan; mixed with supplements B and C) supplemented with 10 μM Y-27632 (Wako, Osaka, Japan) and 0.25 μg/cm^2^ purified laminin-511 fragment (iMatrix-511 silk; Matrixome, Osaka, Japan) was prepared [36]. The plating density was 2,500 cells per cm^2^. The cBA-hiPSCs were single-cell passaged every 6–8 days using 0.5 × TrypLE Select (TrypLE Select (Gibco, Thermo Fisher, Waltham, MA) diluted 1:1 with 0.5 mM EDTA solution) or just 0.5 mM EDTA solution (Nacalai Tesque, Kyoto, Japan). The medium was changed every other day with StemFit AK02N medium (Ajinomoto; mixed with supplements B and C) from the next day of the passage.

### Quantification of remaining EBNA1 sequence in genomic DNA

Genomic DNA was extracted from cBA-hiPSCs at passage numbers 5–15 using DNeasy Blood & Tissue (Qiagen, Venlo, NL). Genomic DNA samples (10 ng) were used as a template to detect the remaining episomal vectors using quantitative PCR (QPCR). The template DNA samples were mixed with Power SYBR Green PCR Master Mix (Thermo Fisher Scientific) and primer sets. QPCR was performed with QuantStudio 3 real-time PCR system (Thermo Fisher Scientific) with default conditions. Copy numbers were calculated from the standard curves of several amounts of synthesized DNA oligos’ corresponding EBNA1 sequence. The primer sets for EBNA1 are listed in Table 2.

**Table 2:** Reagent information

	Antibodies used for immunocytochemistry/flow cytometry/western blot			
	Antibody	Dilution	Company cat #	RRID
Pluripotency marker	Goat anti-OCT3/4	1:200	R and D Systems, Cat# AF1759	RRID:AB_354975
Pluripotency marker	Rabbit anti-NANOG	1:500	ReproCELL Incorporated, Cat# RCAB004P-F,	RRID:AB_1560380
Pluripotency marker	DyLight 550 Mouse anti-SSEA-4	1:125	Stemgent, Cat# 09–0097	RRID:AB_2784538
Pluripotency marker	Alexa Fluor 488 Mouse anti-TRA-1–60	1:125	BioLegend, Cat# 330,613	RRID:AB_2295395
Differentiation marker (Ectoderm)	Mouse anti-TUJ1	1:250	R and D Systems, Cat# MAB1195	RRID:AB_357520
Differentiation marker (Mesoderm)	Mouse anti-SMA	1:250	R and D Systems, Cat# MAB1420	RRID:AB_262054
Differentiation marker (Endoderm)	Mouse anti-AFP	1:200	R and D Systems, Cat# MAB1368	RRID:AB_357658
Secondary antibody for immunocytochemistry	Donkey anti-goat IgG Alexa Flour 546	1:200	Thermo Fisher Scientific, Cat# A-11056	RRID:AB_2534103
Secondary antibody for immunocytochemistry	Goat anti-Rabbit IgG Alexa Flour 555	1:500	Thermo Fisher Scientific, Cat# A-21428	RRID:AB_2535849
Secondary antibody for immunocytochemistry	Donkey anti-mouse IgG Alexa Fluor 488	1:500	Thermo Fisher Scientific, Cat# A-21202	RRID:AB_141607
	Primers			
	Target	Size of band	Forward/reverse primer (5′-3′)	
Mycoplasma detection	Nested-PCR, 1st step PCR (MCGpF11/MCGpR1)	350–850 bp	ACACCATGGGAG(C/T)TGGTAAT/ CTTC(A/T)TCGACTT(C/T)CAGACCCAAGGCAT	
Mycoplasma detection	Nested-PCR, 2nd step PCR (R16–2/MCGpR21)	200–750 bp	GTG(C/G)GG(A/C)TGGATCACCTCCT/ GCATCCACCA(A/T)A(A/T)AC(C/T)CTT	
Episomal vector detection	EBNA1 (genomic qPCR)	61 bp	ATCAGGGCCAAGACATAGAGATG / GCCAATGCAACTTGGACGTT	

### Immunocytochemistry and flow cytometry

Immunocytochemistry and flow cytometry were performed following our previous studies [37–42] at passage number 5–15. For immunocytochemistry, cells were fixed with 4% paraformaldehyde (Wako) and washed with phosphate-buffered saline (PBS). They were permeabilized with 0.1% Triton X-100 (Wako) in PBS and blocked with 1% bovine serum albumin (BSA; Wako) in PBS. These cell samples were incubated with primary antibodies at 4 ºC overnight. After washing these samples three times with PBS, they were incubated with secondary antibodies at room temperature for 1 h. Following three additional PBS washes, the nuclei were stained using Fluoro-KEEPER Antifade Reagent Non-Hardening Type with DAPI (Nacalai Tesque). Images of the cells were captured using a BZ-X800 fluorescence microscope (Keyence). For flow cytometry, the cells were dissociated using Accutase (Nacalai Tesque), resuspended in a custom buffer (0.5% EDTA in PBS supplemented with 1% FBS), and incubated for 1 h with antibodies. The stained cells were analyzed using an SH800S cell sorter and its accompanying software (Sony). The primary and secondary antibodies used in this study are listed in Table 2.

### In vitro* three-germ-layer differentiation assay by forming embryoid bodies (EBs)*

EB formation assay was performed with our previous protocol with minor modifications [37–42]. Briefly, 1.2 × 10^6^ cells were suspended in StemFit AK02N (supplemented with 12 μL of 10 mM Y-27632 solution) into a V-shape 96-well plate. From day 2, these cell aggregates were cultured in DMEM high glucose (Nacalai Tesque) supplemented with 10% fetal bovine serum (Biosera) (EB medium) for 8 days and plated on 0.1w/v% gelatin solution (WAKO)-coated plate in the EB medium for another 8 days. On day 16, these samples were fixed for immunocytochemistry.

### In vivo three-germ-layer differentiation assay by forming teratomas

All animal experiments were approved by the Animal Experimentation Committee at the RIKEN Tsukuba Institute and performed according to the committee’s guiding principles and the “Guide for the Care and Use of Laboratory Animals” published by the National Institutes of Health. Teratomas were formed following our previous studies [37–42]. Briefly, 1.0 × 10^6^ cells were suspended in 50% Matrigel solution (Matrigel (Corning): Stem Fit AK02N medium (Ajinomoto) with 10 µM Y-27632 (Wako) = 1:1) and injected into NSG mice. Teratomas were collected after around 3 months. Differentiation was validated by hematoxylin–eosin (HE) staining (performed at GenoStaff, Tokyo, Japan).

### Virtual karyotyping (CNV/CGH microarray)

Virtual karyotyping assays were performed following our previous study [37–42]. Genomic DNA samples were extracted from hiPSCs at passage numbers 5–15 or PBMCs with DNeasy Blood & Tissue (Qiagen, Venlo, NL). Virtual karyotyping was performed with a GeneChip Scanner 3000 (Thermo Fisher Scientific) using KaryoStat Assay Arrays (Thermo Fisher Scientific) and analyzed with Chromosomal Analysis Suite (ChAS) software (Thermo Fisher Scientific).

### Mycoplasma tests

Indirect DNA fluorescent staining and nested PCR were performed on cBA-hiPSC samples. The cBA-hiPSC culture medium was tested for mycoplasma contamination by staining with bisBenzimide H 33258 (Sigma-Aldrich) after 5–6 days of co-culture with VERO cells (RCB0001, RIKEN BRC Cell Bank), which served as mycoplasma infection indicator cells. DNA samples were extracted and analyzed using nested PCR at passage number 10. For the PCR, AmpliTaq Gold 360 DNA Polymerase (Thermo Fisher Scientific) was used. The same thermocycling conditions were applied for both PCRs: an initial denaturation at 95 °C for 10 min, followed by 30 cycles of thermocycling (30 s at 95 °C, 2 min at 55 °C, and 2 min at 72 °C) with a final extension at 72 °C for 5 min, and a hold at 4 °C. The PCR products were analyzed via electrophoresis on 2% agarose gel and stained with ethidium bromide. The primers used for the PCR are listed in Table 2.

### WGS analysis

Genomic DNA samples from these cBA-hiPSCs were used for whole genome sequence analysis. These genomic DNA samples were fragmented using an ultrasonicator. The fragmented DNA is end-repaired and an A (adenine) is added to the 3ʼ end, and the entire long adapter sequence is ligated. Purification and size selection are then performed. PCR-free library was generated with Illumina DNA PCR-free Prep for these samples. Whole genome sequencing was used to produce around 600 M reads (~ 90 Gb data) from 150 × 2 paired-end (PE150) reads for each sample with Illumina NovaSeq X Plus 25B. Sequence data were analyzed using CLC genomics workbench, version 20. With this software, “quality check (QC) for sequence reads”, “trim reads”, “map reads to reference”, “local realignment”, “basic variant detection”, “amino acid changes”, “annotate with overlap information”, “annotate from known variants”, “filter with control samples”, and “export variant list in csv form” were performed on each sample. Exported variant lists were analyzed with Excel for Mac (ver. 16.89.1) using “autofilter” and “countif” functions. As healthy control hiPSCs, 1383D6 (HPS1006 in RIKEN cell bank) [36] and HiPS-NB1RGB (HPS5067) [43] lines were used.

## Data Availability

The WGS data are available in the NBDC human database as research ID: hum0486. The authors confirm that the other data supporting the findings of this study are available within the article.
